# Continuous monitoring with wearables in multiple sclerosis reveals an association of cardiac autonomic dysfunction with disease severity

**DOI:** 10.1177/20552173221103436

**Published:** 2022-06-01

**Authors:** Marc Hilty, Pietro Oldrati, Liliana Barrios, Tamara Müller, Claudia Blumer, Magdalena Foege, PHRT consortium, Christian Holz, Andreas Lutterotti

**Affiliations:** 27243 University and University Hospital of Zürich, Department of Neurology, Zürich, 8091, Switzerland; 31018 ETH Zürich, Department of Computer Science, Zürich, 8092, Switzerland; 27243 University and University Hospital of Zürich, Department of Neurology, Zürich, 8091, Switzerland; 31018 ETH Zürich, Department of Computer Science, Zürich, 8092, Switzerland; 27243 University and University Hospital of Zürich, Department of Neurology, Zürich, 8091, Switzerland

**Keywords:** multiple sclerosis, progressive, autonomic nervous system, wearable, heart rate variability, cardiac autonomic dysfunction

## Abstract

**Background:**

Dysfunction of the autonomic nervous system is common in multiple sclerosis patients, and probably present years before diagnosis, but its role in the disease is poorly understood.

**Objectives:**

To study the autonomic nervous system in patients with multiple sclerosis using cardiac autonomic regulation measured with a wearable.

**Methods:**

In a two-week study, we present a method to standardize the measurement of heart rate variability using a wearable sensor that allows the investigation of circadian trends. Using this method, we investigate the relationship of cardiac autonomic dysfunction with clinical hallmarks and subjective burden of fatigue and autonomic symptoms.

**Results:**

In 55 patients with multiple sclerosis and 24 healthy age- and gender-matched controls, we assessed the cumulative circadian heart-rate variability trend of two weeks. The trend analysis revealed an effect of inflammation (*P* = 0.0490, SMD = -0.5466) and progressive neurodegeneration (*P* = 0.0016, SMD = 1.1491) on cardiac autonomic function. No association with subjective symptoms could be found.

**Conclusions:**

Trend-based heart rate variability measured with a wearable provides the opportunity for unobtrusive long-term assessment of autonomic functions in patients with multiple sclerosis. It revealed a general dysregulation in patients with multiple sclerosis.

## Introduction

Multiple sclerosis (MS) is a chronic inflammatory disease of the central nervous system (CNS), characterized by multifocal inflammation and widespread neurodegeneration. Both these pathological processes drive the course of the disease and add to the clinical, radiological, and pathological heterogeneity of MS.^1^ Patients with MS (pwMS) are affected by a broad spectrum of neurological disabilities, including motor dysfunction, sensory symptoms, visual problems, impairment in coordination, cognition, fatigue and symptoms of the autonomic nervous system. The autonomic nervous system (ANS), consisting of the sympathetic and parasympathetic nervous systems, regulates involuntary body functions like heart rate (HR), respiration, gastrointestinal, bladder, and sexual function.^2^

Symptoms of ANS dysfunction, like bladder and sexual dysfunction, are among the symptoms with the most significant impact on the quality of life of MS patients.^3^ Moreover, autonomic dysfunction (AD) is present as early as ten years before an MS diagnosis is established, making it the most prominent symptom in the prodromal phase of MS.^4^ Much less is known about cardiac autonomic dysfunction (cAD) in MS and its role in the disease.^5^ A recent meta-analysis revealed a high prevalence of impairment in the sympathetic or the parasympathetic nervous system in pwMS.^6^ In progressive forms of the disease, up to 84% of patients show signs of cAD associated with spinal atrophy.^7^ Still, one-third of patients with subjective AD are asymptomatic during the head-tilt test^8^

Research on cAD in MS has been hampered by the lack of a consensus definition of dysautonomia, different test batteries, and frequently relying on single time-point measurements as opposed to long-term measurements. Understanding cAD in MS provides the opportunity to intervene in key pathophysiological processes of the disease, based on the important role of the ANS in inflammation and immune regulation. The vagus nerve, which mediates parasympathetic effector functions, plays an important role in regulating inflammation through the gut-brain axis and triggers immunoregulatory responses in target organs.^9^ Moreover, sympathetic fibers modulate immune responses through interaction with lymph nodes, leading to the sympathetic nervous system’s suppressing role in CNS-autoimmunity.^10,11^ Hence, assessing the ANS provides key insights into the disease, from the earliest stages of MS (i.e. the prodromal phase), to evaluating disease progression and inflammation.

A common way to assess AD is by measuring cAD by investigating inter-beat-intervals (IBIs) and calculating heart rate variability (HRV) metrics. Both sympathetic and parasympathetic fibers regulate the millisecond differences between beats and have been previously associated with ANS domain dysfunctions.^12^ It has been shown that HRV conveys similar information on cAD as more involving autonomic test batteries such as the Ewing battery.^13–16^

Wearable devices, such as smartwatches, allow heartbeat detection on par with standard ECG measures and provide an easy, non-invasive, long-term observation of HRV, which is needed for unobtrusive assessments in a chronic disease like MS.^17^

In this prospective study, we employed wearable sensors to detect and follow cAD in the long term. We developed a novel approach to analyze trends in HRV over the circadian period, providing a new perspective on HRV analysis and the role of cAD in MS. Finally, we assessed the impact of cAD on subjective symptoms in MS and its association with the two key pathophysiological processes, namely inflammation, and neurodegeneration.

## Methods

### Participants and procedures

This prospective study included patients diagnosed with MS aged 18 or older without concomitant diseases. Patients were recruited at the neuroimmunology outpatient clinic of the University Hospital of Zurich, Switzerland, between 29 November 2019 and 29 July 2021 and provided informed written consent. Healthy controls were recruited from hospital staff as well as family and friends of the investigators, and included after exclusion of chronic illness, regular intake of medication, presence of fatigue or autonomic symptoms. They followed the same protocol as patients. The study protocol was reviewed and approved by the Cantonal Ethics Committee of Zurich and uploaded to kofam.ch (SNCTP000003494). This study was conducted per the declaration of Helsinki, and results are reported according to the STROBE guidelines for cross-sectional trials.^18^

After inclusion, participants completed two validated questionnaires, the fatigue scale for motor and cognitive functions (FSMC) and the abbreviated Composite Autonomic Symptom Score (COMPASS-31).^19,20^ Demographic information, disease state and history were extracted from the clinical information system at the beginning of participation. An inflammatory disease state was defined as either radiological activity (GD + enhancing lesion, new T2 lesion, progressive lesion) or clinical relapse according to Lublin *et al*. within the past 12 months.^21^ Further on, when referring to inflammatory disease state we are using this definition of disease activity. Additionally, we further differentiated inflammation in radiological activity within the past year.

A progressive disease state was defined by neurological deterioration without a relapse event, according to Lublin *et al*. independent of the usual disease course, meaning deteriorating symptoms in an RRMS patients (previously also referred to progressive-relapsing MS) also lead to inclusion in this group.^21^ Further on we refer to progressive patients to all patients showing objective deterioration of symptoms independently of the current disease label.

The ARMSS, as a measure of disease severity, was calculated based on the EDSS at study inclusion.^22^ Clinical routine 3-Tesla MRI images on Phillips, Siemens and a GE scanner for T1, T2, and Flair3D sequences have been exported and processed according to Cerri *et al*. using FreeSurfer 7.0.^23^

Over two weeks, each participant wore a wearable sensor device that is CE-certified for heart-rate detection (Everion, Biofourmis™, medical device class IIa) and was given two sensors to allow continuous monitoring over a 24h period. Raw IBI measures of the photoplethysmography sensor and magnitude of movement (sampled at 1Hz frequency) of the participants were collected. The sensor devices used in this study were previously validated for consistency with a gold-standard Holter ECG device.^17^

### Data processing

The IBIs were checked for artifacts based on the method Berntson *et al*. proposed.^24^ Artifacts were removed, and missing IBIs were linearly interpolated. Subsequently, the data was divided into non-overlapping 5-min segments. Segments that, due to artifact correction, contained more than four interpolated heartbeats in a row were discarded as they are to be considered unreliable.^24^ Furthermore, we excluded all 5-min segments with excessive activity (more details can be found in the supplements). To calculate HRV metrics based on the IBIs, we followed the recommendations of the HRV Task Force for time domain (RMSSD, SDNN, pNN20, pNN50), frequency domain (HF, LF) and nonparametric domain (SD1, SD2).^12^ For each valid segment, metrics for time, frequency, and nonlinear domains were calculated using the open-source Python library *pyHRV*.^25^ According to Ciccone *et al*. and Antali *et al*., most metrics are omitted due to redundancy or due to evidence of them being prone to artifacts, or artificial noise, leaving SDNN, SD1, and SD2 as metrics for evaluation.^26,27^

In a recent publication, Natarajan *et al*. presented normative values for PPG sensor-based HRV features of more than 8 million Fitbit® users.^28^ We divided the computed metrics by the provided age, sex, and time-of-day specific normative values, obtaining percentage scores..^28,29^ These represent the percentage deviation of the measured value from the reference, mitigating the influence of age, sex, and time-of-day differences in group comparisons (also see Figure S2). Next, wake and sleep events were manually labeled based on the accelerometer and HR data and were used to normalize the time of day.

**Figure 2.**
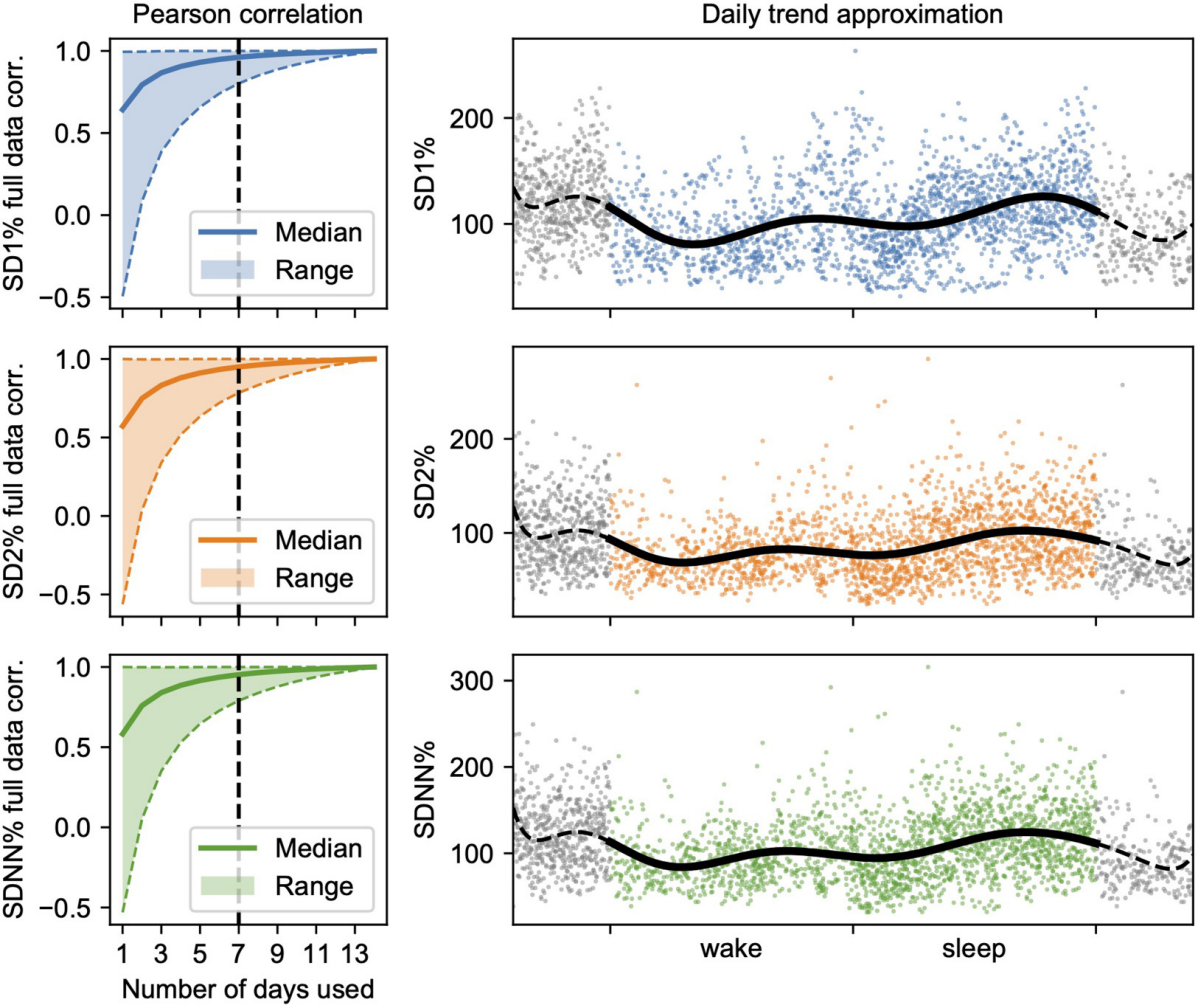
Left: Pearson correlation of the median from the ten approximated trend segments using all possible combinations of available days compared to the full data for all three metrics. The approximations quickly converge, showing that one week of data collection may be sufficient. Right: An example of trend approximation (line) for one patient using the super-imposed daily data (dots) for each metric. Grey dots represent sleep and wake data replicated to the margins to avoid spurious values in the polynomial fit.

At this point, for each HRV metric, we only have single measurements computed from 5-min IBI segments. For each patient, we then super-imposed all the available segments collected during the study. To approximate the real HRV trend of the participants, we fitted 10th degree polynomial regressions for each HRV metric using the least squares method. The resulting models estimate the relationship between the normalized time of the day and the HRV, as shown in Figure 2. In the context of this work, having such an approximation is useful to obtain a smooth estimation of the trend for further analysis and for visualization purposes (more details can be found in the supplements). Subsequently, the resulting trends were split into ten segments (time-windows), of which we calculated the median values. Using an approximated trend calculated from consecutive days allows us to compensate for missing data due to artifacts. We tested the stability of this approach by comparing the estimated time-windows when using only subsets of the available data. Group differences were investigated by applying A) an ordered search over all possible time**-**windows, and B) based on the hypothesis of an adaptive disorder, approximated using the difference between the values of two time-windows (referred to with Δ). We computed the AUC for inflammatory activity within 12 months, progressive disease state, EDSS (threshold ≥ 3), ARMSS (threshold>4), the COMPASS-31 (threshold ≥ 17, as commonly used in diabetic neuropathy) and the FSMC (threshold ≥ 65, for severe fatigue provided through the questionnaire) and chose the best performing time-windows for our statistical analysis.^19,30^ The outline of the data processing pipeline can be seen in the supplemental Figure S3. Data is available on request by contacting the authors.

### Statistical analysis

For descriptive statistics, Chi-Square and Mann-Whitney-U tests were applied for categorical and continuous data, respectively. We calculated both the monotone (Spearman *r_S_*) and linear (Pearson *r_P_*) relationships between variables. We considered α<0.05 significant and employed Benjamini-Hochberg as post-hoc adjustment for multiple testing.^31^ To calculate the AUC confidence intervals we used DeLong's method.^32^ Finally, effect sizes and 95% CIs were reported using the standardized mean difference (SMD). We check if the HRV metrics are confounded by gender, age, or concomitant medications (comprehensive list in the supplemental Table S1) for all the resulting best windows and report any significant findings. Details on the software environment and the libraries used in the analysis can be found in the supplements.

## Results

### Participants

**Figure 1.**
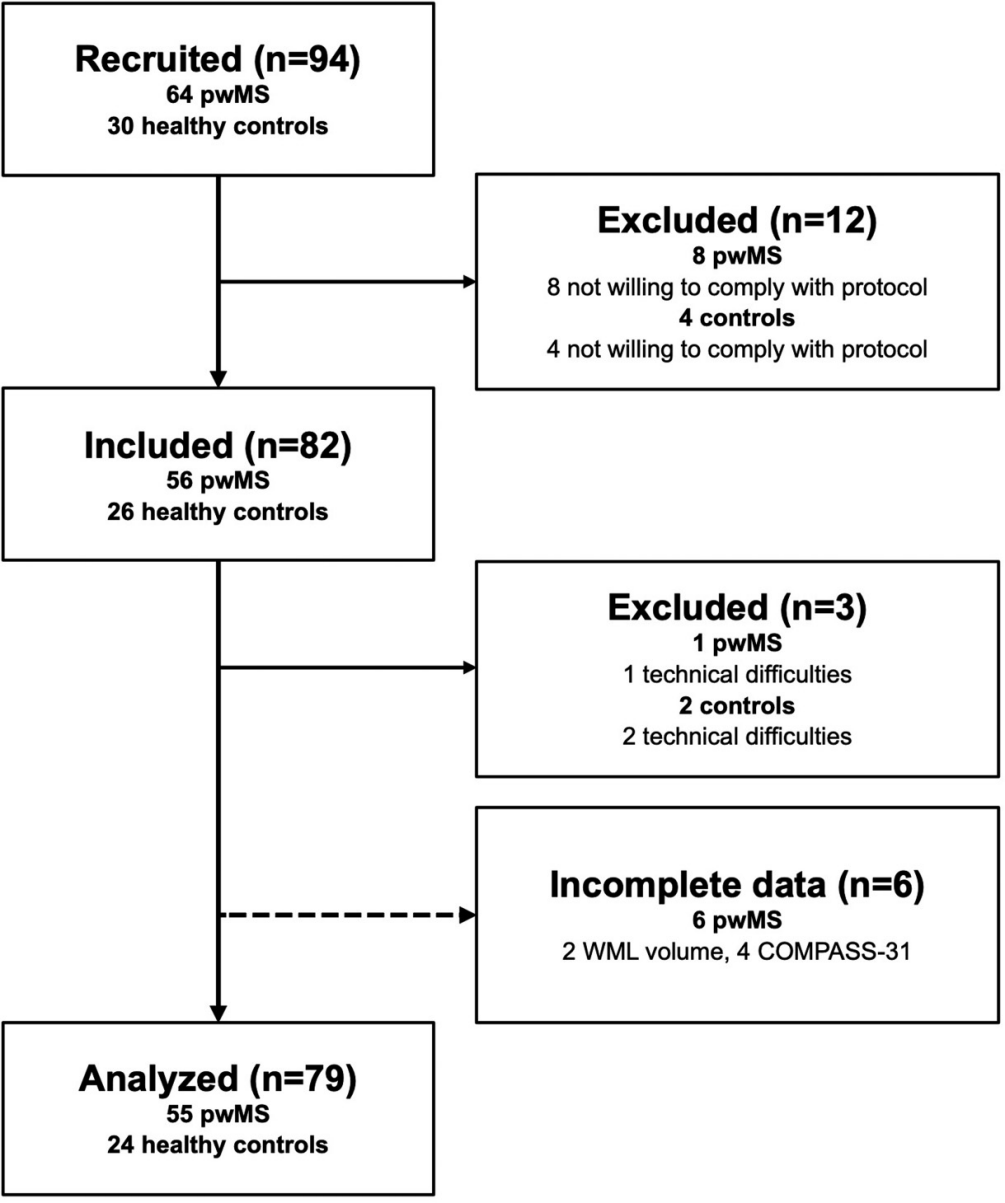
Flow chart of the recruitment process and overview of excluded participants.

We’ve included 79 participants (55 patients, 24 controls) as seen in Figure 1. There is no evidence for age or gender differences (Table 1). Patients had a mean disease duration of 8.77 years (SD:7.79, minimum:0, maximum:28 years) and mostly a relapsing-remitting (RRMS) disease course (46 of 55 patients). A significant age difference between progressive and relapsing patients was found. The mean study duration was 13.380 (minimum:7, maximum:14, SD:1.311) days. Of the RRMS group 17 (30.9%) patients experienced a clinically confirmed relapse in the past year, and 5 (9.0%) showed progressive deterioration of disability. (69.1%) had a stable disease course under disease-modifying treatment. Of the 9 patients labeled as progressive MS (PMS), 3 had a primary-progressive (33.3%) and 6 (66.6%) a secondary-progressive disease course.

**Table 1.** Demographic characteristics of the study population.

		Control	Patient	P-Value
Number		24	55	
Age, mean (SD)		33.5 (10.6)	36.8 (9.5)	0.200
Ethnicity, n(%)	Asian	3 (12.5)		0.019
	Caucasian	18 (75.0)	51 (94.4)	
	Hispanic	1 (4.2)		
	Middle-Eastern	2 (8.3)	3 (5.6)	
Gender, n (%)	m	11 (45.8)	20 (36.4)	0.588
	w	13 (54.2)	35 (63.6)	
MS type, n (%)	PMS		9 (16.4)	
	RRMS		46 (83.6)	
EDSS, mean (SD)			2.2 (1.4)	
Disease duration, mean (SD)			6.7 (6.3)	
Inflam. activity, n (%)	no		38 (69.1)	
	yes		17 (30.9)	
Progressive state, n (%)	no		41 (74.5)	
	yes		14 (25.5)	
DMT, n (%)	None		8 (14.5)	
	Dimethylfumarat		6 (10.9)	
	Natalizumab		12 (21.8)	
	Rituximab		4 (7.3)	
	Ocrelizumab		15 (27.3)	
	Ozanimod		2 (3.6)	
	Siponimod		1 (1.8)	
	Teriflunomide		1 (1.8)	
	aHSCT		6 (10.9)	
FSMC, mean (SD)	total		53.0 (21.8)	
	cognitive		26.2 (11.6)	
	motoric		26.8 (11.1)	
COMPASS-31, mean (SD)			16.9 (8.6)	
Concomittant medication, n (%)	no		35 (63.6)	
	yes		20 (36.4)	

Data are mean (SD) or n (%). MS: multiple sclerosis; PMS: progressive multiple sclerosis; RRMS: relapsing-remitting multiple sclerosis; COMPASS-31: Composite Autonomic Symptom Score; EDSS: expanded disability status score; FSMC: Fatigue Score for motor functions and cognition; aHSCT: autologous hematopoetic stem cell transplantation; DMT: disease modifying therapy; Inflam. activity is defined as confirmed clinical relapse or GD + enhancing lesion in the past 12 months. Progressive state is defined as confirmed clinical progression based on Lublin *et al*.^17^ Concomitant medication is defined as non-DMT therapies with a known influence on heart-rate, a list can be found in the supplemental Table S1.

### Reliability of HRV trend approximation

Analyzing the HRV segments obtained from all possible combinations of available days reveals that the results quickly become correlated with the values estimated using the complete data, as seen in Figure 2. Using seven days of data, we found *r_P_* = 0.9616 (range: [0.8024, 0.9999]), *r_P_* = 0.9495 (range: [0.7846, 0.9998]), and *r_P_* = 0.9524 (range: [0.7901, 0.9998]) for SD1%, SD2% and SDNN% respectively. This suggests that measurement periods of ≥ 7 days are sufficient and that the approximated trends are stable.

Furthermore, we compared nonlinear-domain metrics with frequency-domain measures. The correlation between SD1% and HF% is *r_P_* = 0.9611, while the correlation between the other pair of metrics is *r_P_* = 0.8859. Hence, SD1% and SD2% follow similar trends as HF% and LF%, respectively. Given the more reliable normative data available for nonlinear-domain metrics and their superior reliability in PPG measurements, using SD1% and SD2% is the better choice.^27^

### Associations of CAD with clinical characteristics

**Figure 3.**
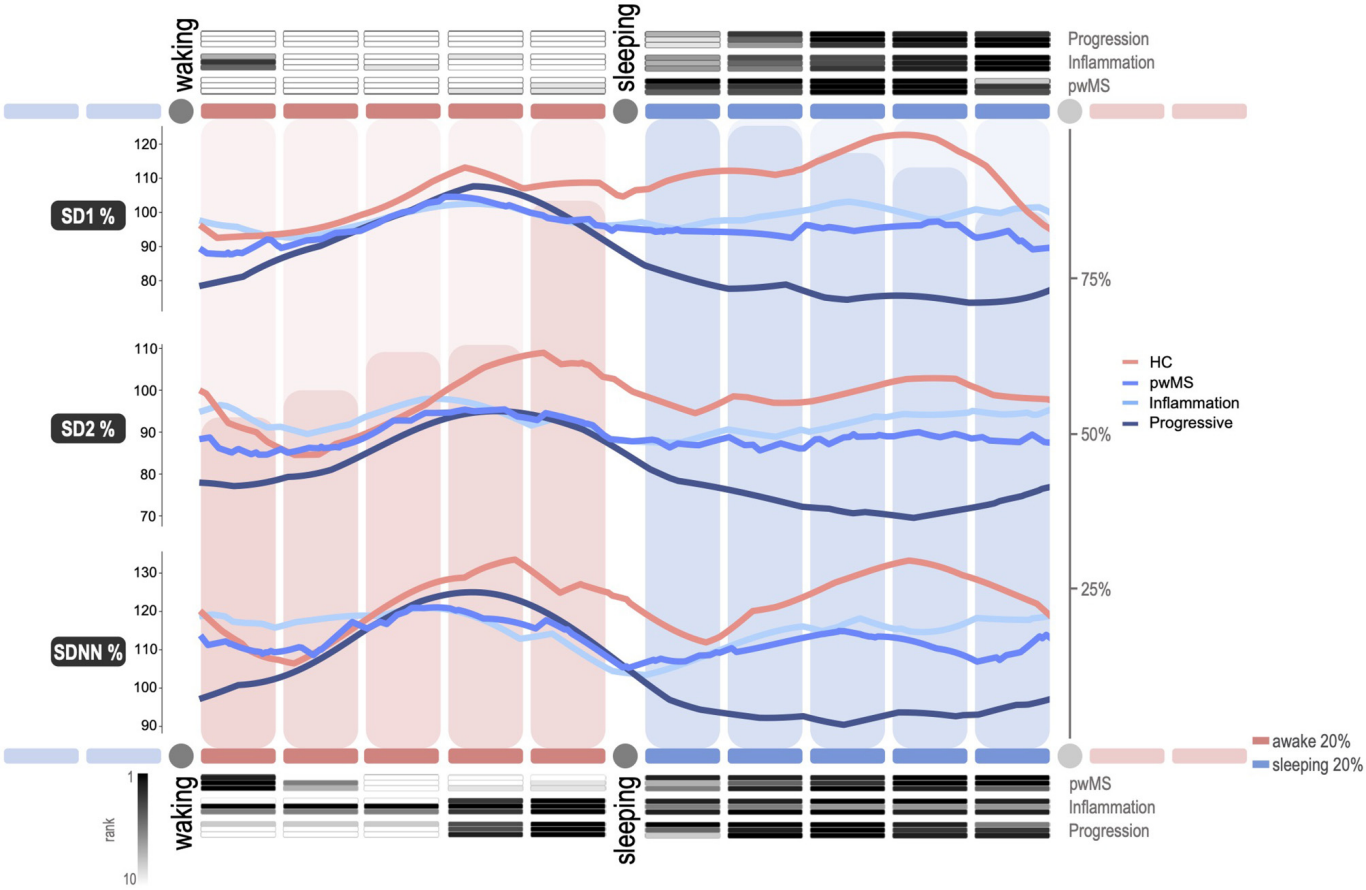
A standardized day starting with waking up in the morning and ending with waking up the day after represented by ten segments (20%), five during awake and five during sleep. The median of the approximated HRV trends of participants groups is shown in the line diagram. The histogram corresponds to the total data availability per segment. The top heat map shows optimal single time windows, the bottom one is the optimal windows for adaptive assessment, ranking the top 10 best AUCs. HC refers to healthy controls, pwMS to patients with multiple sclerosis. During awake hours either early morning or late evening shows differences in the HRV metrics between subgroups, meanwhile signals are converging during a majority of the daytime. Patients with a progressive disease course show a substantially different circadian adaptation, predominantly during sleep.

The circadian trends, as seen in Figure 3, allow us to infer differences in all three measures between pwMS and controls. Table 2 shows the best single time window discriminating pwMS from healthy controls (*P* = 0.0333, SMD = 0.6705, CI:[0.1774, 1.1594]). In Table 3, the best time windows for adaptive, proportional changes in HRV is shown. In this case, the difference between 0–20% of wake and 40–60% is significantly different between pwMS and controls (*P* = 0.0016, SMD = 0.8235, CI: [0.3243, 1.3178]).

**Table 2.** Single time windows discriminating patient characteristics.

Group	Time window	Metric	AUC	95% CI
pwMS	40%-80% night	SD2%	0.6712	0.5378, 0.8046
Inflammation	80%-100% night	SD2%	0.6626	0.5167, 0.8086
Radiological activity	80%-100% night	SD2%	0.655	0.4749, 0.8352
Clinical activity	0%-100% night	SD2%	0.7381	0.5531, 0.9231
Progression	40%-100% night	SD2%	0.7544	0.5916, 0.9171
EDSS	60%-80% night	SD2%	0.7087	0.5464, 0.871
ARMSS	80%-100% night	SDNN%	0.6381	0.4927, 0.7836
COMPASS-31	40%-60% night	SD2%	0.7163	0.5699, 0.8628
COMPASS-31 pwMS	20%-40% day	SD1%	0.6273	0.4736, 0.781
Severe FSMC fatigue	40%-80% day	SD2%	0.6772	0.5056, 0.8489

Optimized time windows and HRV metric based on the AUC to differentiate between patient characteristics: pwMS differentiated from healthy controls (55/79), pwMS with recent inflammatory activity (24/55), pwMS with current radiological activity (12/55), pwMS with current clinical activity (6/55), pwMS with objectified progressive disease (14/55), pwMS with EDSS ≥ 3 (18/55), pwMS with ARMSS ≥ 4 (37/55), pwMS with COMPASS-31 > 17 compared to healthy controls (26/50), pwMS with COMPASS-31 ≥ 17 compared to pwMS wih COMPASS-31 < 17 (26/51) and pwMS with FSMC total score ≥ 65 compared to pwMS without fatigue (21/39).

**Table 3.** Adaptative difference between time windows discriminating patient subgroups.

Group	Time windows	Metric	AUC	95% CI
pwMS	0%-20% day and 40%-60% night	ΔSDNN%	0.7402	[0.6221, 0.8582]
Inflammation	80%-100% day and 40%-100% night	ΔSD1%	0.6707	[0.5236, 0.8178]
Radiological activity	80%-100% day and 60%-80% night	ΔSD1%	0.7403	[0.5564, 0.9242]
Clinical activity	0%-80% day and 20%-60% night	ΔSD1%	0.6497	[0.3663, 0.933]
Progression	80%-100% day and 20%-60% night	ΔSDNN%	0.777	[0.6448, 0.9092]
EDSS	60%-100% day and 40%-80% night	ΔSDNN%	0.7477	[0.6146, 0.8809]
ARMSS	0%-20% night and 40%-80% night	ΔSDNN%	0.6742	[0.5126, 0.8358]
COMPASS-31	60%-80% night and 80%-100% night	ΔSD1%	0.7628	[0.6282, 0.8975]
COMPASS-31 pwMS	40%-80% night and 80%-100% night	ΔSD1%	0.6658	[0.5146, 0.8169]
Severe FSMC fatigue fafatfatigue	40%-80% night and 80%-100% night	ΔSD1%	0.6587	[0.4827, 0.8348]

Optimized difference between two time windows and HRV metric based on the AUC to differentiate between patient characteristics: pwMS differentiated from healthy controls (55/79), pwMS with recent inflammatory activity (24/55), pwMS with current radiological activity (12/55), pwMS with current clinical activity (6/55), pwMS with objectified progressive disease (14/55), pwMS with EDSS ≥ 3 (18/55), pwMS with ARMSS ≥ 4 (37/55), pwMS with COMPASS-31 > 17 compared to healthy controls (26/50), pwMS with COMPASS-31 ≥ 17 compared to pwMS wih COMPASS-31 < 17 (26/51) and pwMS with FSMC total score ≥ 65 compared to pwMS without fatigue (21/39).

The following comparisons were made between patient subgroups. An inflammatory disease state cannot be differentiated using a single segment (*P* = 0.0947, SMD = -0.5055, CI:[-1.0446, 0.0383]), but shows an association with concomitant medications (*r_P_* = −0.3489, P = 0.0271, CI: [-0.5622, −0.0921]) and age (*r_P_* = -0.3489, P = 0.0271, CI: [-0.5622, −0.0921]). However, using the adaptation a significant difference of recent inflammatory activity can be reported (*P* = 0.0490, SMD = -0.5466, CI:[-1.0871, −0.0012]), here we see no confounding factors. Radiological activity showed very similar results: No difference is found with single windows and being the same window as inflammation the same association with medication and age was found. The adaptation during 80–100% of wake and 60–80% of sleep is significantly different (*P* = 0.0355, SMD = -0.7905, CI:[-1.4442, −0.1297]) in patients with radiological activity.

Progressive disease shows a vastly different trend with a significant difference in both single measure (*P* = 0.0148, SMD = 0.7994, CI:[0.1705, 1.4213]) and adaptive difference (*P* = 0.0016, SMD = 1.1491, CI:[0.4995, 1.7890]). However, the single windows are confounded by age (*r_P_* = −0.3335, P = 0.0385, CI: [-0.5501, −0.0748]), which is not the case for adaptive differences.

To differentiate between patients with low (<3) or moderate-to-high EDSS ( ≥ 3), the best single window is 60–80% of sleep, which showed a significant difference (*P* = 0.0389, SMD = 0.6163, CI:[0.0383, 1.1888]). Furthermore, significant Spearman and Pearson correlations were found between EDSS and HRV measurements (*r_S_* = -0.3775, *P* = 0.0135, CI:[-0.5843, −0.1247] and *r_P_* = -0.3502, *P* = 0.0263, CI:[-0.5632, −0.0936]) and unsurprisingly also age (*r_P_* = -0.3434, *P* = 0.0308, CI:[-0.5579, −0.0859]). The windows with the strongest proportional HRV difference are 60–100% of the day and 40–80% of sleep (*P* = 0.0034, SMD = 0.9333, CI:[0.3388, 1.5197]). Also in this case, correlations with EDSS (*r_S_* = -0.3781, *P* = 0.0073, CI:[-0.5847, −0.1253] and *r_P_* = -0.3492, *P* = 0.0103, CI:[-0.5624, −0.0924]) and age (*r_P_* = -0.3241, *P* = 0.0237, CI:[-0.5427, −0.0643]) were found.

Grouping with the ARMSS gave us similar windows to the EDSS. There are no significant differences or correlations with single time windows and an association with medication can be made (*P* = 0.0231, SMD = 0.5816, CI: [0.0186, 1.1393]). However, adaptative differences showed a significant difference (*P* = 0.0436, SMD = 0.5604, CI:[-0.0153, 1.1311]), but no correlations between the ARMSS and HRV trends were significant. Associations between HRV and WML volume can be found in supplemental Table S2. The supplemental Table S3 summarizes all the results in the best time-windows for these groups.

### Assessing subjective symptoms

**Figure 4.**
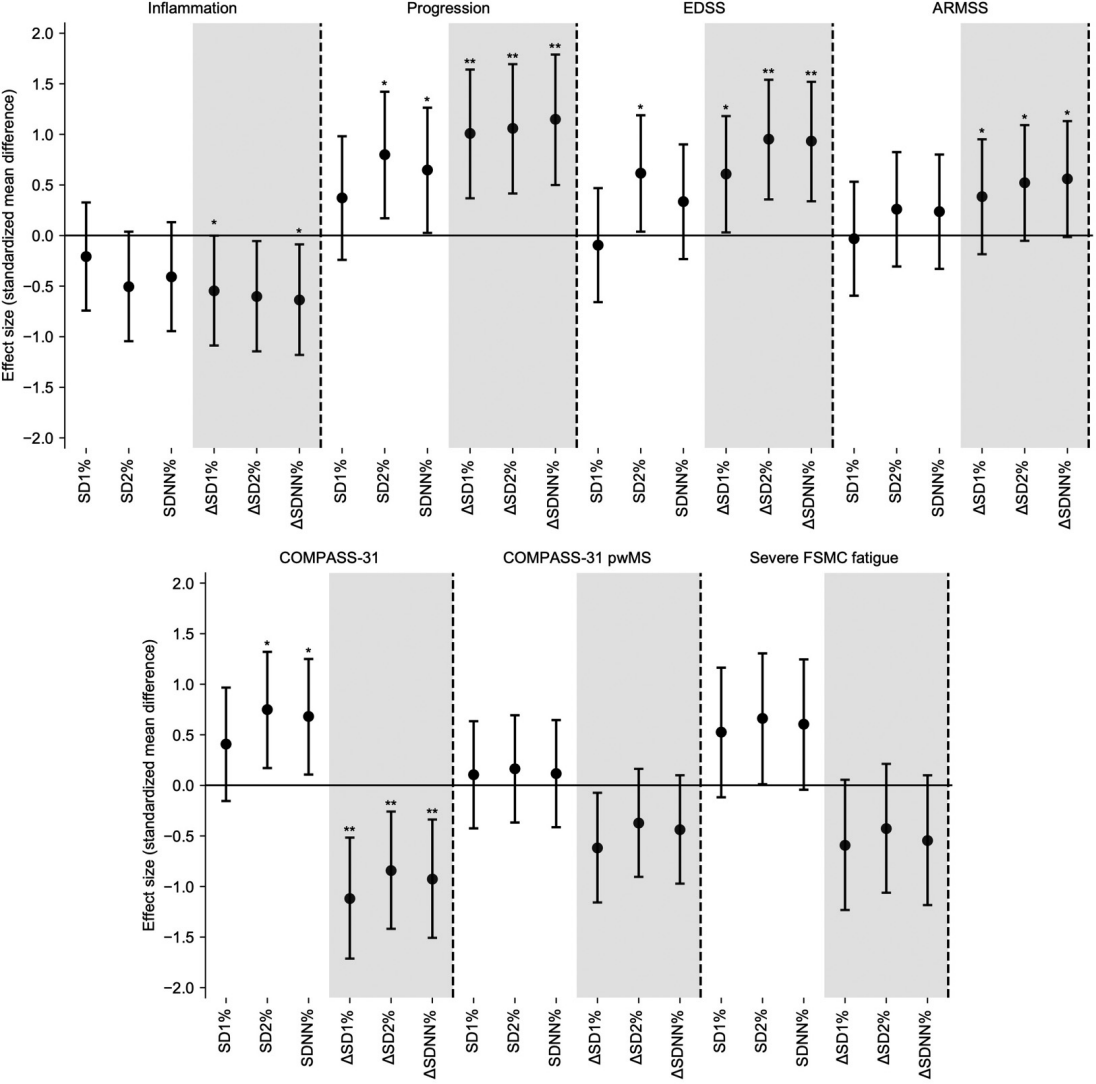
Effect size of difference in pwMS per metric displayed as standardized mean difference with 95% CI. Mann-Whitney-U test was applied and * signifies a p-value <0.05, ** a p-value <0.01, after Benjaminin-Hochberg correction. COMPASS-31 results are displayed both as differences between HC and subjective AD and within patients with or without subjective AD. Other effects where performed only within pwMS.

Subjective AD is commonly reported along with fatigue in pwMS. Hence, we tried to associate HRV and subjective autonomic symptoms based on the COMPASS-31 and FSMC questionnaires (also see Figure 4). As seen in Figure 3, the pwMS with subjective AD show the strongest difference from healthy controls during the 40–60% window in the night using SD2% (*P* = 0.0261, SMD = 0.7492, CI:[0.171, 1.3201]), confounded by medication (*P* = 0.0228, SMD = 0.5307, CI: [-0.0557, 1.1119]). No correlation was found between the COMPASS-31 score and the single segments. Furthermore, concomitant medications seem to confound the HRV measures (*P* = 0.0104, SMD = 0.5457, CI: [-0.0159, 1.1023]). The adaptive proportional change in HRV between the 60–80% and 80–100% of the night showed a significant difference between the groups (*P* = 0.0014, SMD = -1.1196 CI:[-1.7127, −0.5165]), here no effect of medication is found, however a gender association (*P* = 0.0096, SMD = 0.9831, CI: [0.3649, 1.5923]) in this window. Moreover, we can neither report a correlation between HRV and the questionnaire's score, nor a difference in patients with and without subjective ANS symptoms or between non- and severely fatigued patients according to the FSMC. The supplemental Table S4 summarizes all the results in the best time-windows for these groups. Furthermore, the supplemental Figure S1 illustrates the median HRV trends of the groups not previously shown in Figure 3. Finally, the supplemental figure S4 shows the cross-validity of the best time-windows for the different groups as measured by the AUC.

## Discussion

In this study, we present a new approach for analyzing the cardiac autonomic nervous system in the context of MS. Both sympathetic and parasympathetic dysfunctions have been described in pwMS with variable results.^33^ However, a clear understanding of the role of cAD in MS was hampered by the heterogeneity of study designs, methodologies, and inconsistencies of related findings.^34^ As a key finding, we show that the analysis of HRV, based on the adaptation of the autonomic system throughout the circadian rhythm, is a promising method to quantify cAD in MS and allows new insights into disease-related changes. In addition, using this novel method, we can deduce specific patterns of circadian adaptation during different stages of the disease.

A circadian variation in the control of HR and therefore HRV has been described previously in adults and children, showing relevant fluctuations in cardiac autonomic function during sleep.^35,36^ Our results are consistent with these studies, confirming a fluctuation of HRV values over 24h. However, we demonstrate that a continuous measurement of at least seven days is required to establish a robust trend to assess the changes of HRV throughout the circadian rhythm. Measurements over shorter periods are limited by the variability in daily HRV and may have contributed to the heterogeneous results observed in previous studies on cAD in MS, which relied on short-term or even single-time-point measurements.^34^ It is difficult to overcome these effects even by standardizing the measurement time due to inter-individual heterogeneity.

So far, it is unclear whether cAD in MS is related to sympathetic or parasympathetic dysfunction or both. In healthy controls, circadian fluctuation has been considered to be related to a sympathetic withdrawal rather than parasympathetic activity.^35,36^ Our study is the first to recognize autonomic dysfunction in pwMS in the context of circadian differences. Assessing a general trend instead of individual measurements provides several benefits: 1) it is more robust towards outliers as compared to single time windows, 2) temporal variations in HRV are being captured reliably, and 3) circadian fluctuations of HRV can be quantified and do not act as confounding factor.

Our results show a strong association of cAD with a progressive disease state in pwMS, for disability (EDSS), and disease severity (ARMSS). This association has been described previously and was considered to be linked to axonal loss.^7^ However, not all studies came to this conclusion, highlighting the need to control for activities and trend detection in assessing long-term HRV.^37,38^ In this study, a progressive disease state is characterized by a reduced circadian adaptation of the ANS, most prominently during sleep and a generally reduced HRV.

A different effect on cAD can be observed in patients with inflammatory disease activity. Investigation of the trend from evening to the end of the night showed a significant effect in SDNN% and SD1%. While HCs generally showed a decrease in the evening, followed by an increase in SDNN% and SD1%, an inflammatory disease state failed to show this adaptation and presented less variance across the day.

Previous work in pwMS showed that primarily morning windows were used to assess HRV, and an association with inflammatory disease activity was shown.^39^ Our analysis reveals that even though high measures in the morning are present, the circadian adaptation from almost normal values throughout the day transforms into a drop during the night, mainly in the vagally associated measure SD1%. One could hypothesize that the relative change in SD2%, as a measure of sympathetic activity, is lower, suggesting SD2% overactivity during the night and overall, less variability during the day than in HCs.

Within MS patients, the progressive group behaved differently from patients with inflammatory disease activity. Adamec *et al*. report a predominantly parasympathetic dysfunction in progressive patients.^40^ Interestingly, Flachenecker *et al*. hypothesized that the sympathetic nervous system might be dysregulated in pwMS due to its close relationship with inflammatory activity, while parasympathetic dysfunction might primarily result from structural damage in the CNS.^39^ This connection is reflected in a relation of SD1% with MRI white matter lesion volume and a weaker signal in the mainly sympathetic SD2% arm of the ANS in progressive patients.

Altogether, our data suggests that pwMS experience reduced variability in circadian HRV. This is particularly true in case of high cerebral lesion volume and in progressive disease, which is most strongly associated with SD1%.

Even though we could not find within-patient differences in subjective AD measured by the COMPASS-31 score, there is a significant difference between HCs and pwMS with regard to subjective AD, when using our markers of cAD. This suggests that the presence cAD is associated with the disease, but is not directly related to subjective symptoms. A shortcoming of this study is the heterogeneity of the study population, which might obscure a scaling effect for subjective symptoms of AD.

Future studies should also include quality of life assessments to explore further connections of subjective AD, fatigue, and MS-related quality of life. The brain MRI scans used in the study were acquired during clinical routine. Although all scans came from the same MRI center, they were not based on a standardized protocol. Hence, we suggest a standardized acquisition protocols for future studies to investigate the relation of structural damage in the CNS and cAD. Ideally, a quantification of lesion load in the spinal cord would allow to assess a possible link between spinal cord damage and cAD.

Altogether, the current results suggest easy applicability and transferability of this novel method of HRV analysis to other devices and study setups. A better understanding of the role of the ANS in MS offers a wide range of opportunities in MS care, ranging from a potential application as a biomarker for the earliest stages of the disease or as a target for intervention through immuno-regulation and symptomatic therapies.

The advantage of a wearable is that it can potentially be worn for months, and many FDA and EMA certified products are already available on the market. Thus, longitudinal observation of patients may allow new objective measurements of an important symptom domain. However, there is a need for larger cohorts to confirm the results and establish fixed time windows for assessing cAD in pwMS. The latter would provide the opportunity to standardize the HRV parameters when using them in different studies in the future.

## Supplemental Material

sj-docx-1-mso-10.1177_20552173221103436 - Supplemental material for Continuous monitoring with wearables in multiple sclerosis reveals an association of cardiac autonomic dysfunction with disease severity Click here for additional data file. Supplemental material, sj-docx-1-mso-10.1177_20552173221103436 for Continuous monitoring with wearables in multiple sclerosis reveals an association of cardiac autonomic dysfunction with disease severity by Marc Hilty, Pietro Oldrati, Liliana Barrios, Tamara Müller, Claudia Blumer, Magdalena Foege, PHRT consortium, Christian Holz and Andreas Lutterotti in Multiple Sclerosis Journal – Experimental, Translational and Clinical

sj-jpg-2-mso-10.1177_20552173221103436 - Supplemental material for Continuous monitoring with wearables in multiple sclerosis reveals an association of cardiac autonomic dysfunction with disease severity Click here for additional data file. Supplemental material, sj-jpg-2-mso-10.1177_20552173221103436 for Continuous monitoring with wearables in multiple sclerosis reveals an association of cardiac autonomic dysfunction with disease severity by Marc Hilty, Pietro Oldrati, Liliana Barrios, Tamara Müller, Claudia Blumer, Magdalena Foege, PHRT consortium, Christian Holz and Andreas Lutterotti in Multiple Sclerosis Journal – Experimental, Translational and Clinical

sj-jpg-3-mso-10.1177_20552173221103436 - Supplemental material for Continuous monitoring with wearables in multiple sclerosis reveals an association of cardiac autonomic dysfunction with disease severity Click here for additional data file. Supplemental material, sj-jpg-3-mso-10.1177_20552173221103436 for Continuous monitoring with wearables in multiple sclerosis reveals an association of cardiac autonomic dysfunction with disease severity by Marc Hilty, Pietro Oldrati, Liliana Barrios, Tamara Müller, Claudia Blumer, Magdalena Foege, PHRT consortium, Christian Holz and Andreas Lutterotti in Multiple Sclerosis Journal – Experimental, Translational and Clinical

sj-jpg-4-mso-10.1177_20552173221103436 - Supplemental material for Continuous monitoring with wearables in multiple sclerosis reveals an association of cardiac autonomic dysfunction with disease severity Click here for additional data file. Supplemental material, sj-jpg-4-mso-10.1177_20552173221103436 for Continuous monitoring with wearables in multiple sclerosis reveals an association of cardiac autonomic dysfunction with disease severity by Marc Hilty, Pietro Oldrati, Liliana Barrios, Tamara Müller, Claudia Blumer, Magdalena Foege, PHRT consortium, Christian Holz and Andreas Lutterotti in Multiple Sclerosis Journal – Experimental, Translational and Clinical

sj-jpg-5-mso-10.1177_20552173221103436 - Supplemental material for Continuous monitoring with wearables in multiple sclerosis reveals an association of cardiac autonomic dysfunction with disease severity Click here for additional data file. Supplemental material, sj-jpg-5-mso-10.1177_20552173221103436 for Continuous monitoring with wearables in multiple sclerosis reveals an association of cardiac autonomic dysfunction with disease severity by Marc Hilty, Pietro Oldrati, Liliana Barrios, Tamara Müller, Claudia Blumer, Magdalena Foege, PHRT consortium, Christian Holz and Andreas Lutterotti in Multiple Sclerosis Journal – Experimental, Translational and Clinical

sj-docx-6-mso-10.1177_20552173221103436 - Supplemental material for Continuous monitoring with wearables in multiple sclerosis reveals an association of cardiac autonomic dysfunction with disease severity Click here for additional data file. Supplemental material, sj-docx-6-mso-10.1177_20552173221103436 for Continuous monitoring with wearables in multiple sclerosis reveals an association of cardiac autonomic dysfunction with disease severity by Marc Hilty, Pietro Oldrati, Liliana Barrios, Tamara Müller, Claudia Blumer, Magdalena Foege, PHRT consortium, Christian Holz and Andreas Lutterotti in Multiple Sclerosis Journal – Experimental, Translational and Clinical

sj-docx-7-mso-10.1177_20552173221103436 - Supplemental material for Continuous monitoring with wearables in multiple sclerosis reveals an association of cardiac autonomic dysfunction with disease severity Click here for additional data file. Supplemental material, sj-docx-7-mso-10.1177_20552173221103436 for Continuous monitoring with wearables in multiple sclerosis reveals an association of cardiac autonomic dysfunction with disease severity by Marc Hilty, Pietro Oldrati, Liliana Barrios, Tamara Müller, Claudia Blumer, Magdalena Foege, PHRT consortium, Christian Holz and Andreas Lutterotti in Multiple Sclerosis Journal – Experimental, Translational and Clinical

sj-docx-8-mso-10.1177_20552173221103436 - Supplemental material for Continuous monitoring with wearables in multiple sclerosis reveals an association of cardiac autonomic dysfunction with disease severity Click here for additional data file. Supplemental material, sj-docx-8-mso-10.1177_20552173221103436 for Continuous monitoring with wearables in multiple sclerosis reveals an association of cardiac autonomic dysfunction with disease severity by Marc Hilty, Pietro Oldrati, Liliana Barrios, Tamara Müller, Claudia Blumer, Magdalena Foege, PHRT consortium, Christian Holz and Andreas Lutterotti in Multiple Sclerosis Journal – Experimental, Translational and Clinical

sj-docx-9-mso-10.1177_20552173221103436 - Supplemental material for Continuous monitoring with wearables in multiple sclerosis reveals an association of cardiac autonomic dysfunction with disease severity Click here for additional data file. Supplemental material, sj-docx-9-mso-10.1177_20552173221103436 for Continuous monitoring with wearables in multiple sclerosis reveals an association of cardiac autonomic dysfunction with disease severity by Marc Hilty, Pietro Oldrati, Liliana Barrios, Tamara Müller, Claudia Blumer, Magdalena Foege, PHRT consortium, Christian Holz and Andreas Lutterotti in Multiple Sclerosis Journal – Experimental, Translational and Clinical

## References

[1] Noseworthy JH Lucchinetti C Rodriguez M , et al. Multiple sclerosis. N Engl J Med 2000; 343: 938–952. 11006371 10.1056/NEJM200009283431307

[2] Jänig W . Autonomic nervous system. In: Schmidt RF Thews G Herausgeber D (eds) Human physiology [internet]. Berlin, Heidelberg: Springer, 1989, pp.S. 333–70. Verfügbar unter.

[3] Cortez MM Nagi Reddy SK Goodman B , et al. Autonomic symptom burden is associated with MS-related fatigue and quality of life. Mult Scler Relat Disord 2015; 4: 258–263. 26008943 10.1016/j.msard.2015.03.007

[4] Disanto G Zecca C MacLachlan S , et al. Prodromal symptoms of multiple sclerosis in primary care. Ann Neurol 2018; 83: 1162–1173. 29740872 10.1002/ana.25247

[5] Findling O Hauer L Pezawas T , et al. Cardiac autonomic dysfunction in multiple sclerosis: a systematic review of current knowledge and impact of immunotherapies. J Clin Med 2020; 9: 335. 10.3390/jcm9020335 PMC7073977 31991711

[6] Racosta JM Kimpinski K Morrow SA , et al. Autonomic dysfunction in multiple sclerosis. Auton Neurosci 2015; 193: 1–6. 26070809 10.1016/j.autneu.2015.06.001

[7] de Seze J Stojkovic T Gauvrit J-Y , et al. Autonomic dysfunction in multiple sclerosis: cervical spinal cord atrophy correlates. J Neurol 2001; 248: 297–303. 11374094 10.1007/s004150170204

[8] Arbogast SD Alshekhlee A Hussain Z , et al. Hypotension unawareness in profound orthostatic hypotension. Am J Med 2009; 122: 574–580. 19486719 10.1016/j.amjmed.2008.10.040

[9] Benarroch EE . Autonomic nervous system and neuroimmune interactions: new insights and clinical implications. Neurology 2019; 92: 377–385. 30651384 10.1212/WNL.0000000000006942

[10] Araujo LP Maricato JT Guereschi MG , et al. The sympathetic nervous system mitigates CNS autoimmunity via β2–adrenergic receptor signaling in immune cells. Cell Rep. 2019; 28: 3120–3130.e5. 31533035 10.1016/j.celrep.2019.08.042

[11] Flachenecker P . Autonomic dysfunction in Guillain-Barré syndrome and multiple sclerosis. J Neurol 2007; 254: II96–I101. 17503142 10.1007/s00415-007-2024-3

[12] Malik M . Heart rate variability: standards of measurement, physiological interpretation, and clinical use: task force of the European society of cardiology and the North American society for pacing and electrophysiology. Ann Noninvasive Electrocardiol 1996; 1: 151–181.

[13] Ewing DJ Martyn CN Young RJ , et al. The value of cardiovascular autonomic function tests: 10 years experience in diabetes. Diabetes Care 1985; 8: 491–498. 4053936 10.2337/diacare.8.5.491

[14] Jirkovská A Boucek P Wu S , et al. Power spectral analysis of heart rate variability in patients with charcot’s neuroarthropathy. J Am Podiatr Med Assoc 2006; 96: 1–8. 16415277 10.7547/0960001

[15] Howorka K Pumprla J Schabmann A . Optimal parameters of short-term heart rate spectrogram for routine evaluation of diabetic cardiovascular autonomic neuropathy. J Auton Nerv Syst 1998; 69: 164–172. 9696273 10.1016/s0165-1838(98)00015-0

[16] Malik M Hnatkova K Huikuri HV , et al. Crosstalk proposal: heart rate variability is a valid measure of cardiac autonomic responsiveness. J Physiol 2019; 597: 2595–2598. 31006862 10.1113/JP277500 PMC6826215

[17] Barrios L Oldrati P Santini S , et al. Evaluating the accuracy of heart rate sensors based on photoplethysmography for in-the-wild analysis. In: Proceedings of the 13th EAI international conference on pervasive computing technologies for healthcare [internet]. New York, NY, USA: Association for Computing Machinery, 2019 [zitiert 9. August 2021], pp.S. 251–61. (PervasiveHealth’19). Verfügbar unter.

[18] von Elm E Altman DG Egger M , et al. The strengthening the reporting of observational studies in epidemiology (STROBE) statement: guidelines for reporting observational studies. J Clin Epidemiol 2008; 61: 344–349. 18313558 10.1016/j.jclinepi.2007.11.008

[19] Penner IK Raselli C Stöcklin M , et al. The fatigue scale for motor and cognitive functions (FSMC): validation of a new instrument to assess multiple sclerosis-related fatigue. Mult Scler Houndmills Basingstoke Engl 2009; 15: 1509–1517. 10.1177/1352458509348519 19995840

[20] Sletten DM Suarez GA Low PA , et al. COMPASS 31: a refined and abbreviated composite autonomic symptom score. Mayo Clin Proc 2012; 87: 1196–1201. 23218087 10.1016/j.mayocp.2012.10.013 PMC3541923

[21] Lublin FD Reingold SC Cohen JA , et al. Defining the clinical course of multiple sclerosis. Neurology 2014; 83: 278–286. 24871874 10.1212/WNL.0000000000000560 PMC4117366

[22] Roxburgh R Seaman SR Masterman T , et al. Multiple sclerosis severity score: using disability and disease duration to rate disease severity. Neurology 2005; 64: 1144–1151. 15824338 10.1212/01.WNL.0000156155.19270.F8

[23] Cerri S Puonti O Meier DS , et al. A contrast-adaptive method for simultaneous whole-brain and lesion segmentation in multiple sclerosis. NeuroImage 2020; 225: 117471. 33099007 10.1016/j.neuroimage.2020.117471 PMC7856304

[24] Berntson GG Quigley KS Jang JF , et al. An approach to artifact identification: application to heart period data. Psychophysiology 1990; 27: 586–598. 2274622 10.1111/j.1469-8986.1990.tb01982.x

[25] Caridade Gomes PM . Development of an open-source Python toolbox for heart rate variability (HRV) [PhD Thesis]. Hochschule für angewandte Wissenschaften Hamburg; 2019.

[26] Ciccone AB Siedlik JA Wecht JM , et al. Reminder: RMSSD and SD1 are identical heart rate variability metrics. Muscle Nerve 2017; 56: 674–678. 28073153 10.1002/mus.25573

[27] Antali F Kulin D Lucz KI , et al. Multimodal assessment of the pulse rate variability analysis module of a photoplethysmography-based telemedicine system. Sensors 2021; 21: 5544. 34450986 10.3390/s21165544 PMC8401087

[28] Natarajan A Pantelopoulos A Emir-Farinas H , et al. Heart rate variability with photoplethysmography in 8 million individuals: a cross-sectional study. Lancet Digit Health 2020; 2: e650–e657. 33328029 10.1016/S2589-7500(20)30246-6

[29] Bonnemeier H Wiegand UKH Brandes A , et al. Circadian profile of cardiac autonomic nervous modulation in healthy subjects. J Cardiovasc Electrophysiol 2003; 14: 791–799. 12890036 10.1046/j.1540-8167.2003.03078.x

[30] Greco C Gennaro FD D’Amato C , et al. Validation of the composite autonomic symptom score 31 (COMPASS 31) for the assessment of symptoms of autonomic neuropathy in people with diabetes. Diabet Med 2017; 34: 834–838. 27990686 10.1111/dme.13310

[31] Benjamini Y Hochberg Y . Controlling the false discovery rate: a practical and powerful approach to multiple testing. J R Stat Soc Ser B Methodol 1995; 57: 289–300.

[32] DeLong ER DeLong DM Clarke-Pearson DL . Comparing the areas under two or more correlated receiver operating characteristic curves: a nonparametric approach. Biometrics 1988; 44: 837–845. 3203132

[33] Habek M Krbot Skorić M . Autonomic nervous system: a key player in prodromal multiple sclerosis? Clin Auton Res 2020; 30: 97–99. 32162038 10.1007/s10286-020-00676-3

[34] Racosta JM Sposato LA Morrow SA , et al. Cardiovascular autonomic dysfunction in multiple sclerosis: a meta-analysis. Mult Scler Relat Disord 2015; 4: 104–111. 25787186 10.1016/j.msard.2015.02.002

[35] Malpas SC Purdie GL . Circadian variation of heart rate variability. Cardiovasc Res 1990; 24: 210–213. 2346954 10.1093/cvr/24.3.210

[36] Massin MM Maeyns K Withofs N , et al. Circadian rhythm of heart rate and heart rate variability. Arch Dis Child 2000; 83: 179–182. 10906034 10.1136/adc.83.2.179 PMC1718415

[37] Reynders T Gidron Y De Ville J , et al. Relation between heart rate variability and disease course in multiple sclerosis. J Clin Med 2020; 9: 3. 10.3390/jcm9010003 PMC7019937 31861312

[38] Damla O Altug C Pinar KK , et al. Heart rate variability analysis in patients with multiple sclerosis. Mult Scler Relat Disord 2018; 24: 64–68. 29957350 10.1016/j.msard.2018.06.012

[39] Flachenecker P Reiners K Krauser M , et al. Autonomic dysfunction in multiple sclerosis is related to disease activity and progression of disability. Mult Scler J 2001; 7: 327–334. 10.1177/135245850100700509 11724449

[40] Adamec I Crnošija L Junaković A , et al. Progressive multiple sclerosis patients have a higher burden of autonomic dysfunction compared to relapsing remitting phenotype. Clin Neurophysiol 2018; 129: 1588–1594. 29885648 10.1016/j.clinph.2018.05.009

