# Supplementary material for: Continuous monitoring with wearables in multiple sclerosis reveals an association of cardiac autonomic dysfunction with disease severity
Source: Mult Scler J Exp Transl Clin. 2022 Jun 1;8(2):20552173221103436. doi: 10.1177/20552173221103436 (PMC9168869; doi:10.1177/20552173221103436)
Supplement: sj-docx-1-mso-10.1177_20552173221103436 - Supplemental material for Continuous monitoring with wearables in multiple sclerosis reveals an association of cardiac autonomic dysfunction with disease severity [file sj-docx-1-mso-10.1177_20552173221103436.docx]

Supplemental Material

**Members of the PHRT consortium**

The following people are part of the personalized health and related technologies (PHRT) consortium funding body and was involved in the planning, execution and monitoring of study procedures and results. Hafen Ernst, Raetsch Gunnar, Perez-Cruz Fernando, Martin Roland, Bignens Serge, Esteban Cristobal, Napolitano Stefano, Salamanca Luis, Gasser Lilian, Braeunlich Gerhard, Descloux Olivier, Drewlow Joshua, Hess Gabriel, Iseli Stefan, Kaiser Carole, Kreutz Alexander, Maffioletti Sergio, Meier Lea, von Kaenel Francois, Zimmermann Marc, Baumann Martina.

**Approximating the HRV trend using polynomial regression**

Previous work has shown that the relationship between time of the day and HRV is nonlinear. (1,2) This is reflected in the normative values published by Natarajan et al. which are used in this work. (3) Hence, to approximate the daily HRV trend, a nonlinear model is needed. Previous work derived circadian rhythms of HRV by fitting cosine curves with the least squares method. (1,2) However, since our HRV metrics have been normalized using normative values which could alter the signal shape, we decided to use a more general method with fewer assumptions on the underlying shape of the trend. Therefore, we opted for polynomial regression, also fitted with the least-squares method. The polynomial degree, the only parameter of such a model, was set to 10 after analyzing the decrease of the residual error between the approximation and the measurements. Having a model that approximates the trends is also useful for visualizations such as in Figure 3 and Figure S1. The statistical analysis in this work is based on the median values of 10 segments (time windows) derived from the approximations. This approach is robust towards outliers that may create erroneous fluctuations in the approximated trend. The result is a further decrease in the risk of overfitting the noise in the data.

**Software environment**

For the analysis we used Python 3.8.10 with the following packages: numpy 1.19.5, pandas 1.2.4, matplotlib 3.4.3, seaborn 0.11.1, scipy 1.7.1, pyhrv 0.4.0, and tableone 0.7.10. Furthermore, we used R version 3.6.3 with the effsize 0.8.1 package. (4)

**Removal of active segment**

To select HRV segments for removal, we looked at their context using the heart rate and magnitude of movement measured by the wearable. In total, we defined 5 different context types. *Resting* is defined as <55% of the participant's maximal heart rate (HR_age_ = 220 BPM - age), with no measurable continuous activity in the accelerometer; *activity* as >55% HR_age_ and sustained movement; *warmup* refers to sustained movement with heart rate <55% HR_age_ before an activity interval; *cooldown* as >55% HR_age_ with no movement after an activity interval; and *stress* as a heart rate increase >55% HR_age_ with no active or adjacent exercise activity segments. (5) To reduce artifacts and better match the sensor validation experiments, for the final analysis we only used IBIs from data segments that were categorized as *resting*. (6)

**Potential Role of spinal cord lesions in cAD**

One must consider that lesions in the spinal cord are associated with a higher risk of disease progression. Simultaneously most pwMS have at least one spinal lesion. Hence, we investigated symptomatic spinal involvement defined by a conservative estimate using at least one radiologically confirmed spinal lesion as well as clinical signs of spinal cord involvement. For symptomatic spinal involvement no difference using single segments was found. However, adaptative differences show a highly significant difference for ΔSD2% between 0-20% and 20-60% during the night (P=0.0006, SMD=1.0545, CI:[0.4828, 1.6174]), with no evidence for potential confounding of age, gender or medication and an AUC of 0.7667 [0.6376, 0.8957]. This pronounced trend in this group can also be observed in figure S1. However, no firm conclusion can be drawn on whether cAD is a direct consequence of lesions in the spinal cord or rather neurodegeneration occurring across different parts of the CNS. In addition, a large fraction of patients without symptomatic spinal cord involvement, had spinal cord lesions on MRI.

**References**

1. Massin MM. Circadian rhythm of heart rate and heart rate variability. Arch Dis Child. 1. August 2000;83(2):179–82.

2. Yang Z, Liu H, Meng F, Guan Y, Zhao M, Qu W, u. a. The analysis of circadian rhythm of heart rate variability in patients with drug-resistant epilepsy. Epilepsy Res. 1. Oktober 2018;146:151–9.

3. Natarajan A, Pantelopoulos A, Emir-Farinas H, Natarajan P. Heart rate variability with photoplethysmography in 8 million individuals: A cross-sectional study. Lancet Digit Health. 2020;2(12):e650–7.

4. Caridade Gomes PM. Development of an open-source Python toolbox for heart rate variability (HRV) [PhD Thesis]. Hochschule für angewandte Wissenschaften Hamburg; 2019.

5. American College of Sports Medicine, Riebe D, Ehrman JK, Liguori G, Magal M. ACSM’s guidelines for exercise testing and prescription. 2018.

6. Barrios L, Oldrati P, Santini S, Lutterotti A. Evaluating the accuracy of heart rate sensors based on photoplethysmography for in-the-wild analysis. In: Proceedings of the 13th EAI International Conference on Pervasive Computing Technologies for Healthcare [Internet]. New York, NY, USA: Association for Computing Machinery; 2019 [zitiert 9. August 2021]. S. 251–61. (PervasiveHealth’19). Verfügbar unter: https://doi.org/10.1145/3329189.3329215
