# Supplementary material for: Continuous monitoring with wearables in multiple sclerosis reveals an association of cardiac autonomic dysfunction with disease severity
Source: Mult Scler J Exp Transl Clin. 2022 Jun 1;8(2):20552173221103436. doi: 10.1177/20552173221103436 (PMC9168869; doi:10.1177/20552173221103436)
Supplement: sj-docx-6-mso-10.1177_20552173221103436 - Supplemental material for Continuous monitoring with wearables in multiple sclerosis reveals an association of cardiac autonomic dysfunction with disease severity [file sj-docx-6-mso-10.1177_20552173221103436.docx]

**Table S1**. Concomitant medication in the study population

| **Medication** | **N** |
| --- | --- |
| Antidepressants | 9 |
| Antihypertensive | 4 |
| Sympathomimetic | 3 |
| Neuroleptic | 3 |
| Psychostimulant | 1 |
| Parasympatholytic | 1 |
