# Supplementary material for: Continuous monitoring with wearables in multiple sclerosis reveals an association of cardiac autonomic dysfunction with disease severity
Source: Mult Scler J Exp Transl Clin. 2022 Jun 1;8(2):20552173221103436. doi: 10.1177/20552173221103436 (PMC9168869; doi:10.1177/20552173221103436)
Supplement: sj-docx-7-mso-10.1177_20552173221103436 - Supplemental material for Continuous monitoring with wearables in multiple sclerosis reveals an association of cardiac autonomic dysfunction with disease severity [file sj-docx-7-mso-10.1177_20552173221103436.docx]

**Table S2**. Correlation between white matter lesions volume and HRV measurements

|  | **Metric** | **Spearman correlation r_S_** | **Pearson correlation r_P_** |
| --- | --- | --- | --- |
| Inflammation | SD1% | -0·2653, [-0·5041, 0·0111], *P* = 0·0599 | -0·1493, [-0·4081, 0·1317], *P* = 0·2957 |
|  | SD2% | -0·3705, [-0·5862, -0·1057], *P* = 0·0223* | -0·2868, [-0·5212, -0·0122], *P* = 0·1210 |
|  | SDNN% | -0·2714, [-0·5089, 0·0045], *P* = 0·0599 | -0·2469, [-0·4892, 0·0308], *P* = 0·1210 |
|  | ΔSD1% | -0·3180, [-0·5458, -0·0465], *P* = 0·0344* | -0·2494, [-0·4912, 0·0282], *P* = 0·0776 |
|  | ΔSD2% | -0·3310, [-0·5559, -0·0610], *P* = 0·0344* | -0·3952, [-0·6049, -0·1343], *P* = 0·0123* |
|  | ΔSDNN% | -0·2829, [-0·5181, -0·0079], *P* = 0·0443* | -0·3550, [-0·5744, -0·0880], *P* = 0·0159* |
| Progression | SD1% | -0·2746, [-0·5115, 0·0011], *P* = 0·0512 | -0·1924, [-0·4444, 0·0879], *P* = 0·1763 |
|  | SD2% | -0·3682, [-0·5845, -0·1031], *P* = 0·0235* | -0·3566, [-0·5756, -0·0898], *P* = 0·0306* |
|  | SDNN% | -0·2847, [-0·5195, -0·0099], *P* = 0·0512 | -0·3070, [-0·5371, -0·0343], *P* = 0·0427* |
|  | ΔSD1% | -0·3471, [-0·5683, -0·0790], *P* = 0·0378* | -0·3580, [-0·5767, -0·0914], *P* = 0·0186* |
|  | ΔSD2% | -0·2446, [-0·4874, 0·0332], *P* = 0·0836 | -0·3310, [-0·5559, -0·0610], *P* = 0·0186* |
|  | ΔSDNN% | -0·2565, [-0·4969, 0·0206], *P* = 0·0836 | -0·3284, [-0·5539, -0·0581], *P* = 0·0186* |
| EDSS | SD1% | -0·2757, [-0·5123, -0·0001], *P* = 0·0502 | -0·1796, [-0·4337, 0·1010], *P* = 0·2074 |
|  | SD2% | -0·3839, [-0·5964, -0·1211], *P* = 0·0163* | -0·3652, [-0·5822, -0·0997], *P* = 0·0252* |
|  | SDNN% | -0·2871, [-0·5214, -0·0125], *P* = 0·0502 | -0·3167, [-0·5447, -0·0450], *P* = 0·0354* |
|  | ΔSD1% | -0·3367, [-0·5603, -0·0673], *P* = 0·0322* | -0·3231, [-0·5497, -0·0521], *P* = 0·0208* |
|  | ΔSD2% | -0·3133, [-0·5421, -0·0413], *P* = 0·0322* | -0·3969, [-0·6062, -0·1362], *P* = 0·0085** |
|  | ΔSDNN% | -0·3004, [-0·5319, -0·0270], *P* = 0·0322* | -0·3818, [-0·5948, -0·1187], *P* = 0·0085** |
| ARMSS | SD1% | -0·2653, [-0·5041, 0·0111], *P* = 0·0599 | -0·1493, [-0·4081, 0·1317], *P* = 0·2957 |
|  | SD2% | -0·3705, [-0·5862, -0·1057], *P* = 0·0223* | -0·2868, [-0·5212, -0·0122], *P* = 0·1210 |
|  | SDNN% | -0·2714, [-0·5089, 0·0045], *P* = 0·0599 | -0·2469, [-0·4892, 0·0308], *P* = 0·1210 |
|  | ΔSD1% | -0·2358, [-0·4802, 0·0425], *P* = 0·0957 | -0·0592, [-0·3294, 0·2199], *P* = 0·6797 |
|  | ΔSD2% | -0·3485, [-0·5694, -0·0807], *P* = 0·0366* | -0·4049, [-0·6121, -0·1455], *P* = 0·0096** |
|  | ΔSDNN% | -0·2740, [-0·5110, 0·0017], *P* = 0·0775 | -0·3368, [-0·5604, -0·0675], *P* = 0·0235* |
| COMPASS-31 | SD1% | -0·1557, [-0·5260, 0·2643], *P* = 0·4677 | -0·2279, [-0·5782, 0·1933], *P* = 0·2841 |
|  | SD2% | -0·3139, [-0·6367, 0·1025], *P* = 0·4057 | -0·3398, [-0·6536, 0·0737], *P* = 0·2481 |
|  | SDNN% | -0·1670, [-0·5344, 0·2535], *P* = 0·4677 | -0·2925, [-0·6225, 0·1257], *P* = 0·2481 |
|  | ΔSD1% | 0·2557, [-0·1647, 0·5974], *P* = 0·5354 | 0·2492, [-0·1715, 0·5930], *P* = 0·6366 |
|  | ΔSD2% | 0·1330, [-0·2857, 0·5091], *P* = 0·5354 | 0·1016, [-0·3147, 0·4851], *P* = 0·6366 |
|  | ΔSDNN% | 0·1757, [-0·2451, 0·5407], *P* = 0·5354 | 0·1236, [-0·2945, 0·5020], *P* = 0·6366 |
| COMPASS-31 pwMS | SD1% | 0·0330, [-0·2448, 0·3058], *P* = 0·8872 | -0·1074, [-0·3719, 0·1734], *P* = 0·5240 |
|  | SD2% | -0·0793, [-0·3473, 0·2007], *P* = 0·8872 | -0·1183, [-0·3814, 0·1626], *P* = 0·5240 |
|  | SDNN% | -0·0204, [-0·2943, 0·2567], *P* = 0·8872 | -0·0913, [-0·3579, 0·1890], *P* = 0·5240 |
|  | ΔSD1% | 0·1826, [-0·0979, 0·4363], *P* = 0·5571 | 0·2094, [-0·0703, 0·4585], *P* = 0·1441 |
|  | ΔSD2% | 0·0842, [-0·1960, 0·3516], *P* = 0·5571 | 0·2075, [-0·0723, 0·4569], *P* = 0·1441 |
|  | ΔSDNN% | 0·1265, [-0·1545, 0·3886], *P* = 0·5571 | 0·2300, [-0·0487, 0·4755], *P* = 0·1441 |
| Severe fatigue | SD1% | -0·0249, [-0·3552, 0·3109], *P* = 0·8870 | -0·1188, [-0·4348, 0·2233], *P* = 0·4966 |
|  | SD2% | -0·1857, [-0·4887, 0·1573], *P* = 0·8564 | -0·1664, [-0·4734, 0·1767], *P* = 0·4966 |
|  | SDNN% | -0·0765, [-0·3995, 0·2635], *P* = 0·8870 | -0·1500, [-0·4603, 0·1929], *P* = 0·4966 |
|  | ΔSD1% | 0·2546, [-0·0859, 0·5419], *P* = 0·4197 | 0·2792, [-0·0596, 0·5603], *P* = 0·1401 |
|  | ΔSD2% | 0·1025, [-0·2389, 0·4214], *P* = 0·5578 | 0·2545, [-0·0861, 0·5418], *P* = 0·1401 |
|  | ΔSDNN% | 0·1585, [-0·1845, 0·4671], *P* = 0·5445 | 0·2754, [-0·0637, 0·5575], *P* = 0·1401 |
| Clinical activity | SD1% | -0·2294, [-0·4750, 0·0493], *P* = 0·1456 | -0·2197, [-0·4670, 0·0595], *P* = 0·1214 |
|  | SD2% | -0·2714, [-0·5089, 0·0045], *P* = 0·1456 | -0·3015, [-0·5328, -0·0282], *P* = 0·0859 |
|  | SDNN% | -0·2067, [-0·4563, 0·0731], *P* = 0·1456 | -0·2680, [-0·5062, 0·0082], *P* = 0·0859 |
|  | ΔSD1% | -0·3465, [-0·5679, -0·0784], *P* = 0·0382* | -0·3268, [-0·5527, -0·0563], *P* = 0·0447* |
|  | ΔSD2% | -0·2168, [-0·4647, 0·0625], *P* = 0·1264 | -0·2644, [-0·5033, 0·0121], *P* = 0·0608 |
|  | ΔSDNN% | -0·2788, [-0·5149, -0·0035], *P* = 0·0713 | -0·3045, [-0·5352, -0·0316], *P* = 0·0447* |
| Radiological activity | SD1% | -0·2653, [-0·5041, 0·0111], *P* = 0·0599 | -0·1493, [-0·4081, 0·1317], *P* = 0·2957 |
|  | SD2% | -0·3705, [-0·5862, -0·1057], *P* = 0·0223* | -0·2868, [-0·5212, -0·0122], *P* = 0·1210 |
|  | SDNN% | -0·2714, [-0·5089, 0·0045], *P* = 0·0599 | -0·2469, [-0·4892, 0·0308], *P* = 0·1210 |
|  | ΔSD1% | -0·3013, [-0·5326, -0·0280], *P* = 0·0334* | -0·2234, [-0·4700, 0·0557], *P* = 0·1151 |
|  | ΔSD2% | -0·3349, [-0·5589, -0·0654], *P* = 0·0334* | -0·4064, [-0·6133, -0·1473], *P* = 0·0093** |
|  | ΔSDNN% | -0·2985, [-0·5304, -0·0249], *P* = 0·0334* | -0·3684, [-0·5846, -0·1033], *P* = 0·0117* |

Correlations and 95% CIs between WML volume and the HRV measurements for each AUC-optimized window. P-values were corrected using Benjiamini-Hochberg.
