# Supplementary material for: Continuous monitoring with wearables in multiple sclerosis reveals an association of cardiac autonomic dysfunction with disease severity
Source: Mult Scler J Exp Transl Clin. 2022 Jun 1;8(2):20552173221103436. doi: 10.1177/20552173221103436 (PMC9168869; doi:10.1177/20552173221103436)
Supplement: sj-docx-8-mso-10.1177_20552173221103436 - Supplemental material for Continuous monitoring with wearables in multiple sclerosis reveals an association of cardiac autonomic dysfunction with disease severity [file sj-docx-8-mso-10.1177_20552173221103436.docx]

**Table S3**. Group differences in optimized windows for clinical characteristics

| **Condition** | **Metric** | **False** | **True** | **P-value** | **SMD [95% CI]** |
| --- | --- | --- | --- | --- | --- |
| Inflammation, n |  | 31 | 24 |  |  |
|  | SD1% | 85 (42.1) | 100 (32.5) | 0.1376 | -0.2081 [-0.7415, 0.3272] |
|  | SD2% | 82 (30.3) | 94 (22.7) | 0.0947 | -0.5055 [-1.0446, 0.0383] |
|  | SDNN% | 101 (39.7) | 118 (31.5) | 0.0947 | -0.4083 [-0.9449, 0.1321] |
|  | ΔSD1% | -9 (24.9) | 4 (33.8) | 0.0490* | -0.5466 [-1.0871, -0.0012] |
|  | ΔSD2% | -7 (19.8) | 0 (20.9) | 0.0541 | -0.6025 [-1.1448, -0.0547] |
|  | ΔSDNN% | -5 (16.7) | 4 (19.2) | 0.0498* | -0.6367 [-1.1803, -0.0874] |
| Progression, n |  | 41 | 14 |  |  |
|  | SD1% | 100 (41.4) | 74 (35.7) | 0.0522 | 0.3721 [-0.2404, 0.9812] |
|  | SD2% | 96 (31.2) | 71 (18.5) | 0.0148* | 0.7994 [0.1705, 1.4213] |
|  | SDNN% | 118 (32.4) | 93 (27.7) | 0.0314* | 0.6476 [0.0257, 1.2637] |
|  | ΔSD1% | 1 (21.8) | -14 (22.5) | 0.0019** | 1.0089 [0.3683, 1.6408] |
|  | ΔSD2% | -5 (19.0) | -18 (8.0) | 0.0016** | 1.0594 [0.4157, 1.6941] |
|  | ΔSDNN% | -4 (18.6) | -19 (10.0) | 0.0016** | 1.1491 [0.4995, 1.7890] |
| EDSS ≥3), n |  | 37 | 18 |  |  |
|  | SD1% | 102 (41.3) | 80 (51.9) | 0.226 | -0.0951 [-0.6582, 0.4688] |
|  | SD2% | 95 (33.3) | 72 (25.7) | 0.0389* | 0.6163 [0.0383, 1.1888] |
|  | SDNN% | 116 (38.5) | 94 (47.7) | 0.1403 | 0.3355 [-0.2329, 0.9007] |
|  | ΔSD1% | 4 (31.2) | -16 (28.9) | 0.0136* | 0.6084 [0.0307, 1.1806] |
|  | ΔSD2% | -4 (18.5) | -17 (9.5) | 0.0035** | 0.9524 [0.3568, 1.5399] |
|  | ΔSDNN% | -3 (18.9) | -14 (14.2) | 0.0034** | 0.9333 [0.3388, 1.5197] |
| ARMSS (≥4), n |  | 18 | 37 |  |  |
|  | SD1% | 99 (21.7) | 88 (51.7) | 0.1539 | -0.0316 [-0.5947, 0.5318] |
|  | SD2% | 95 (25.1) | 86 (31.0) | 0.1539 | 0.2605 [-0.3061, 0.8247] |
|  | SDNN% | 122 (32.1) | 103 (40.1) | 0.1539 | 0.2366 [-0.3296, 0.8005] |
|  | ΔSD1% | 7 (25.7) | 1 (24.1) | 0.0436* | 0.3850 [-0.1847, 0.9512] |
|  | ΔSD2% | 5 (13.8) | -6 (14.9) | 0.0436* | 0.5220 [-0.0523, 1.0915] |
|  | ΔSDNN% | 8 (16.5) | -2 (13.9) | 0.0436* | 0.5604 [-0.0153, 1.1311] |

Overview of study metrics performance for optimized window selected differences. Data is shown as median and IQR with a Mann-Whitney-U test between nonparametric groups with Benjamini-Hochberg post-hoc correction and standardized mean difference to describe the effect size.
