# Supplementary material for: Continuous monitoring with wearables in multiple sclerosis reveals an association of cardiac autonomic dysfunction with disease severity
Source: Mult Scler J Exp Transl Clin. 2022 Jun 1;8(2):20552173221103436. doi: 10.1177/20552173221103436 (PMC9168869; doi:10.1177/20552173221103436)
Supplement: sj-docx-9-mso-10.1177_20552173221103436 - Supplemental material for Continuous monitoring with wearables in multiple sclerosis reveals an association of cardiac autonomic dysfunction with disease severity [file sj-docx-9-mso-10.1177_20552173221103436.docx]

**Table S4**. Group differences in optimizes windows for subjective symptom burden.

|  |  | **False** | **True** | **P-value** | **SMD [95% CI]** |
| --- | --- | --- | --- | --- | --- |
| COMPASS-31 (≥17), n |  | 24 | 26 |  |  |
|  | SD1% | 117 (36.1) | 91 (56.4) | 0.0756 | 0.4081 [-0.1547, 0.9668] |
|  | SD2% | 99 (35.4) | 84 (21.2) | 0.0261* | 0.7492 [0.1710, 1.3201] |
|  | SDNN% | 128 (49.4) | 111 (35.2) | 0.0261* | 0.6815 [0.1069, 1.2494] |
|  | ΔSD1% | -10 (6.5) | 2 (13.9) | 0.0014** | -1.1196 [-1.7127, -0.5165] |
|  | ΔSD2% | -3 (4.9) | 4 (13.3) | 0.0090** | -0.8428 [-1.4185, -0.2590] |
|  | ΔSDNN% | -3 (7.5) | 5 (13.9) | 0.0063** | -0.9270 [-1.5075, -0.3378] |
| COMPASS-31 pwMS (≥17), n |  | 29 | 26 |  |  |
|  | SD1% | 95 (29.6) | 86 (34.9) | 0.2346 | 0.1052 [-0.4250, 0.6345] |
|  | SD2% | 90 (21.4) | 83 (30.4) | 0.2346 | 0.1638 [-0.3672, 0.6933] |
|  | SDNN% | 118 (20.5) | 104 (36.8) | 0.2346 | 0.1165 [-0.4139, 0.6457] |
|  | ΔSD1% | -5 (16.8) | 4 (19.7) | 0.0989 | -0.6185 [-1.1579, -0.0735] |
|  | ΔSD2% | 0 (12.3) | 5 (17.9) | 0.2482 | -0.3726 [-0.9049, 0.1632] |
|  | ΔSDNN% | 0 (12.7) | 6 (17.6) | 0.2482 | -0.4381 [-0.9719, 0.0998] |
| Severe FSMC fat., n |  | 18 | 21 |  |  |
|  | SD1% | 106 (31.1) | 101 (35.5) | 0.2781 | 0.5263 [-0.1179, 1.1637] |
|  | SD2% | 101 (36.3) | 82 (42.6) | 0.183 | 0.6622 [0.0107, 1.3052] |
|  | SDNN% | 128 (41.9) | 110 (52.7) | 0.2578 | 0.6050 [-0.0432, 1.2455] |
|  | ΔSD1% | -2 (14.7) | 8 (19.6) | 0.2209 | -0.5925 [-1.2325, 0.0551] |
|  | ΔSD2% | 1 (9.3) | 4 (22.6) | 0.3749 | -0.4274 [-1.0616, 0.2124] |
|  | ΔSDNN% | 0 (11.1) | 7 (21.9) | 0.3749 | -0.5457 [-1.1838, 0.0995] |

Overview of study metrics performance for optimized window selected differences. Data is shown as median and IQR with a Mann-Whitney-U test between nonparametric groups with Benjamini-Hochberg post-hoc correction and standardized mean difference to describe the effect size.
